# Oxidative Stress-Mediated RUNX3 Mislocalization Occurs Via Jun Activation Domain-Binding Protein 1 and Histone Modification

**DOI:** 10.1007/s12010-024-04944-0

**Published:** 2024-04-29

**Authors:** Kyoung Ah Kang, Mei Jing Piao, Pincha Devage Sameera Madushan Fernando, Herath Mudiyanselage Udari Lakmini Herath, Hye-Jin Boo, Sang Pil Yoon, Jin Won Hyun

**Affiliations:** 1 Jeju Research Center for Natural Medicine, Jeju National University, Jeju, 63243 Republic of Korea; 2 Department of Biochemistry, Jeju National University College of Medicine, Jeju, 63243 Republic of Korea

**Keywords:** Runt domain transcription factor 3, Oxidative stress, Jun activation domain-binding protein 1, Histone modification, Cytoplasmic localization

## Abstract

Runt domain transcription factor 3 (RUNX3) suppresses many different cancer types and is disabled by mutations, epigenetic repression, or cytoplasmic mislocalization. In this study, we investigated whether oxidative stress is associated with RUNX3 accumulation from the nucleus to the cytoplasm in terms of histone modification. Oxidative stress elevated histone deacetylase (HDAC) level and lowered that of histone acetyltransferase. In addition, oxidative stress decreased the expression of mixed lineage leukemia (MLL), a histone methyltransferase, but increased the expression of euchromatic histone-lysine N-methyltransferase 2 (EHMT2/G9a), which is also a histone methyltransferase. Moreover, oxidative stress-induced RUNX3 phosphorylation, Src activation, and Jun activation domain-binding protein 1 (JAB1) expression were inhibited by knockdown of HDAC and G9a, restoring the nuclear localization of RUNX3 under oxidative stress. Cytoplasmic RUNX3 localization was followed by oxidative stress-induced histone modification, activated Src along with RUNX3 phosphorylation, and induction of JAB1, resulting in RUNX3 inactivation.

## Introduction

Runt-related transcription factor 3 (RUNX3), a member of the Runt domain family and a nuclear transcriptional regulator, play important roles in a variety of biological processes, including development, cell proliferation, differentiation, and DNA repair [1, 2]. RUNX3 containing an evolutionarily conserved Runt DNA-binding domain and a C-terminal trans-activating domain functions primarily as a tumor suppressor [3]. RUNX3 is commonly inactive in different types of solid tumors due to epigenetic silencing, mutation, or mislocalization from the nucleus to the cytoplasm [4, 5]. RUNX3 mislocalization from the nucleus to the cytoplasm occurs in various advanced carcinomas, including non-small cell lung cancer, and inactivates tumor suppression, suggesting that nuclear RUNX3 plays a key role in cancer suppression [6–9].

Jun activation domain-binding protein 1 (JAB1), which is part of the COP9 signalosome (CSN), facilitates nuclear chromosomal region maintenance 1 (CRM1) export and proteasome-mediated RUNX3 degradation [10, 11]. In addition, the Src/Stat3 signaling pathway plays a crucial role in controlling JAB1 transcription in breast cancer cells, and treatment with Stat3 and Src-targeting siRNAs noticeably reduces JAB1 promoter function and expression [12].

Src-mediated RUNX3 phosphorylation induces RUNX3 mislocalization in various cancers, including colon cancer [13, 14]. In *Helicobacter pylori*, Src-mediated RUNX3 phosphorylation induces cytoplasmic RUNX3 localization [15]. Several epidemiological studies have demonstrated the occurrence of increased levels of reactive oxygen species (ROS) and oxidative stress markers in the blood of patients with colorectal cancer [16, 17]. In addition, the induction of cytoplasmic RUNX3 retention under hypoxic culture condition in gastric cancer cells occurs via the upregulation of histone deacetylase (HDAC) and histone methyltransferase (HMT), euchromatic histone-lysine N-methyltransferase 2 (EHMT2/G9a) [18]. In a previous study, we demonstrated that oxidative stress-inactivated RUNX3 participates in abnormal cell proliferation via Akt/β-catenin/cyclin D cascade activation in human colon cancer cells [19]. Furthermore, we showed that oxidative stress suppressed RUNX3 expression and promoted translocation to the cytoplasm in colon cancer cells by increasing HDAC level [13, 20]. Oxidative stress is known to play a role in critical changes in nuclear transport, leading to protein mislocalization, which in turn contributes to cancer pathogenesis [21–23]. However, the correlation between RUNX3, HDAC, HMT, and nuclear exporter under oxidative stress remains unknown.

Therefore, in this study, we investigated the correlation between RUNX3, HDAC, HMT, and nuclear exporter to determine the cytoplasmic distribution of RUNX3 in colon cancer cells under oxidative stress.

## Materials and Methods

### Cell Viability Assay

SNU-407, a human colon cancer cell line (Korea Cell Line Bank, Seoul, Republic of Korea), was cultured with various concentrations of H_2_O_2_ for 24 h. After the addition of 3-(4,5-dimethylthiazol-2-yl)-2,5-diphenyltetrazolium bromide (MTT), the absorbance at 540 nm was measured using a scanning multi-well spectrophotometer.

### Intracellular ROS Detection

Cells were treated with 25 µM of 2′,7′-dichlorodihydrofluorescein diacetate (H_2_DCFDA; Molecular Probes, Eugene, OR, USA), a cell-permeant ROS probe, and the fluorescence was detected using a flow cytometer (Becton Dickinson, Mountain View, CA, USA).

### Immunofluorescence Assay

Fixed cells were incubated with an antibody against RUNX3, and followed by treatment with an FITC-conjugated secondary antibody (Santa Cruz Biotechnology, Dallas, TX, USA). The stained cells were observed using a confocal microscope equipped with LSM 510 software.

### Western Blot Analysis

Cell lysate was electrophoresed, transferred, and incubated with relevant primary antibodies, phospho-RUNX3, RUNX3, TATA-binding protein (TBP), actin (Abcam, Cambridge, MA, USA); phospho-Src, Src, histone acetyltransferase 1 (HAT1), G9a (Cell Signaling Technology, Danvers, MA, USA); JAB1, CRM1, HDAC1, and mixed lineage leukemia (MLL) (Santa Cruz Biotechnology). An immunoglobulin G conjugated with horseradish peroxidase (Pierce, Rockford, IL, USA) was used as secondary antibody. Protein bands were identified using an improved chemiluminescence western blot detection kit (Amersham, Little Chalfont, Buckinghamshire, UK).

### Transient Transfection of Small Interfering RNAs (siRNAs)

Cells were transfected with either control siRNA or siRNAs that specifically targeted Src, JAB1, HDAC1, or G9a (referred to as siSrc, siJAB1, siHDAC1, and siG9a, respectively) (Santa Cruz Biotechnology). Transfection was performed using Lipofectamine RNAiMax reagent (Invitrogen, Carlsbad, CA, USA).

### Immunoprecipitation Assay

Cell lysates were incubated with antibodies targeting RUNX3, JAB1, CRM1, IgG, and immune complexes were prepared using protein A/G PLUS beads (Santa Cruz Biotechnology). Specific antibodies were used to identify precipitates by western blot analysis.

### Proximity Ligation Assay (PLA)

Cells were permeabilized by using the mouse/rabbit red starter Duolink kit according to the manufacturer’s instructions (Sigma-Aldrich, Burlington, MA, USA). The cells were then incubated with primary antibodies, JAB1 (1:100), CRM1 (1:100), and RUNX3 (1:100). The cells were then treated with PLA probes and visualized using a confocal microscope and the LSM 510 program.

### Statistical Analysis

All data are presented as mean ± standard error, and the results were subjected to analysis of variance using Tukey’s test. Statistical significance was set at *p* < 0.05.

## Results

### Oxidative Stress Induces RUNX3 Mislocalization Via Histone Modification-Related Proteins

**Fig. 1:**
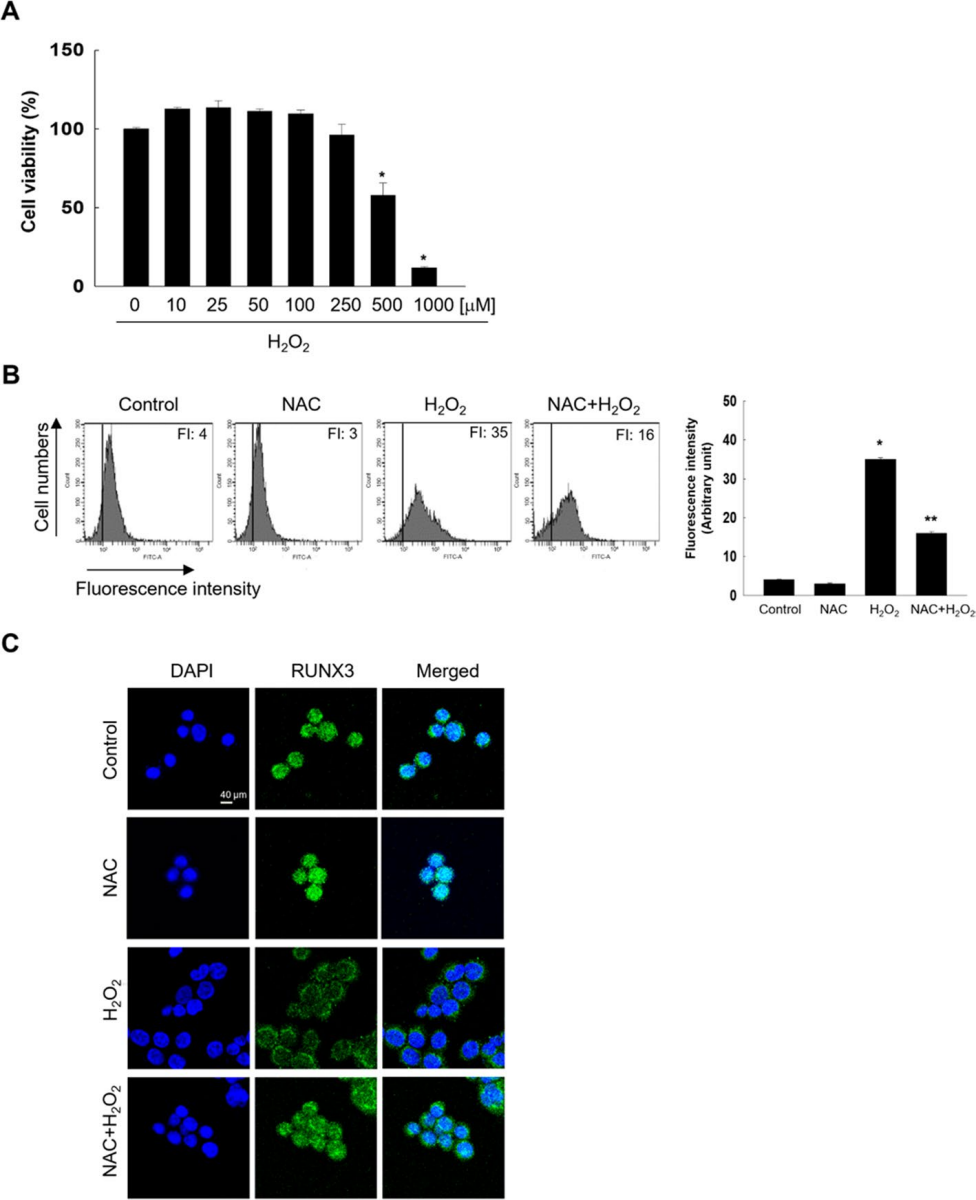

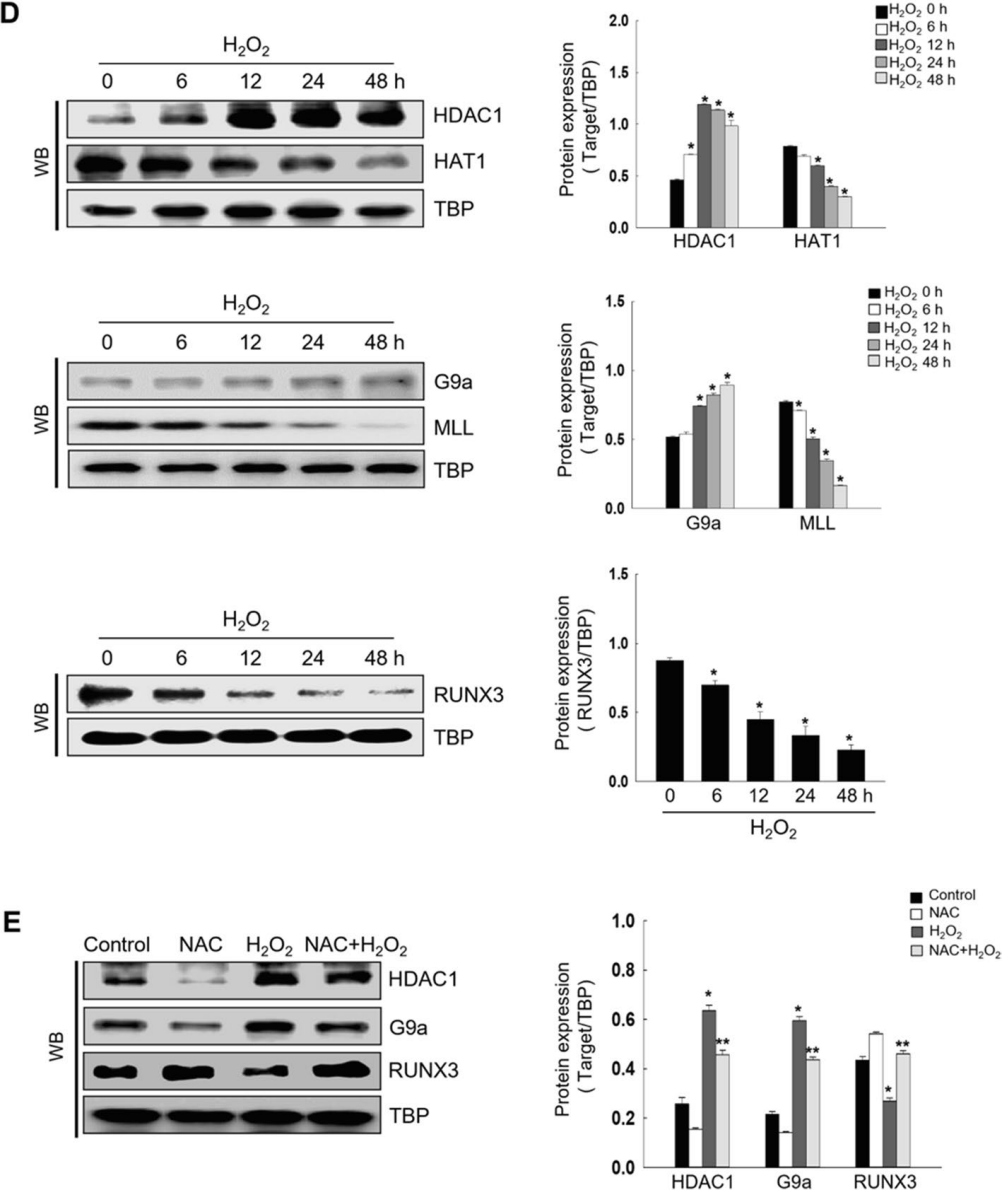

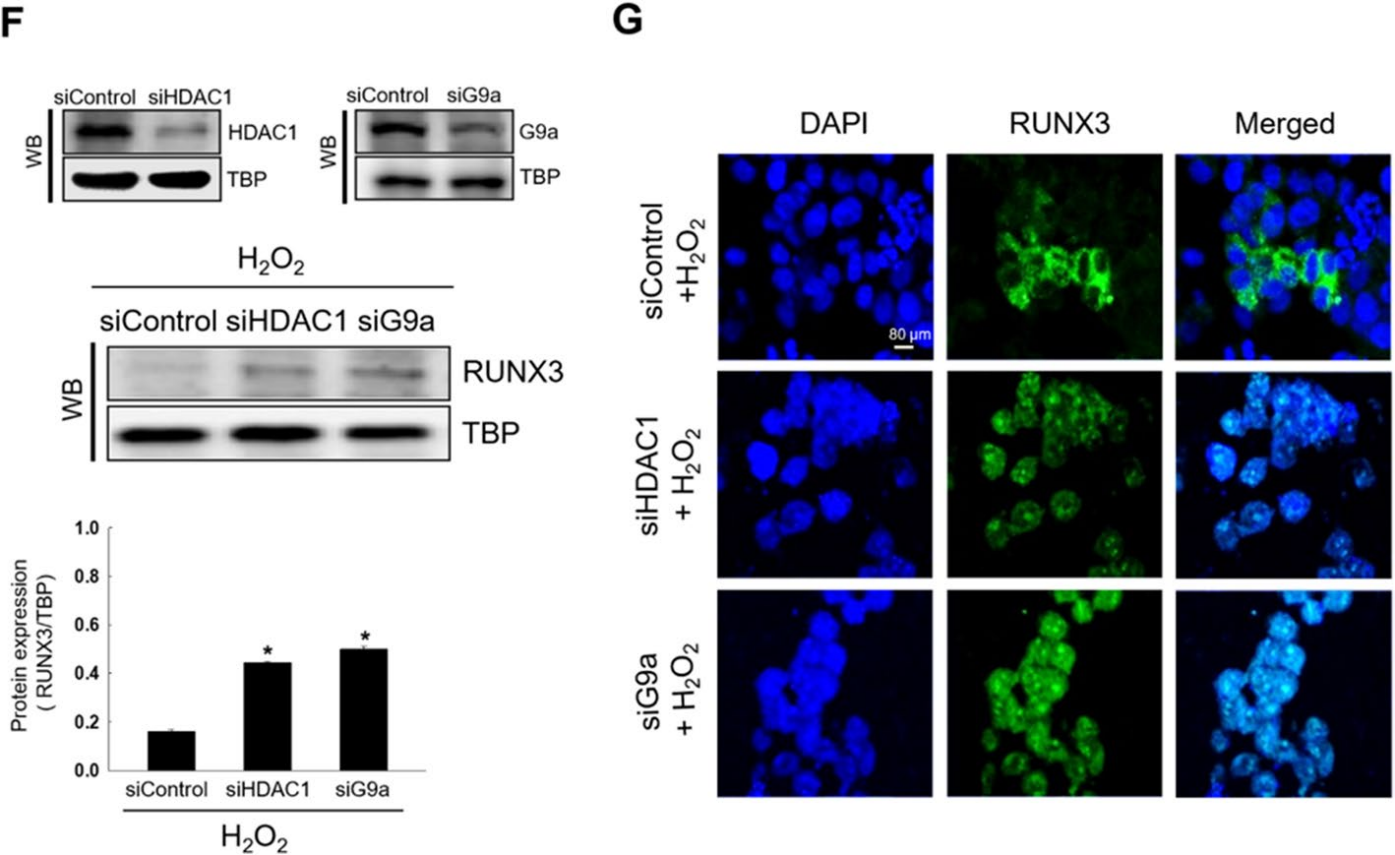
Oxidative stress-induced RUNX3 mislocalization by histone modification-related proteins. **A** Cell viability was assessed using MTT assay. *Significantly different from control cells (*p* < 0.05). Cells were pretreated with 1 mM NAC for 1 h, followed by treatment with H_2_O_2_ for 48 h. **B** ROS levels were determined using flow cytometry after H_2_DCFDA staining. ^*^Significantly different from control cells (*p* < 0.05); ^**^significantly different from H_2_O_2_-treated cells (*p* < 0.05). **C** Confocal microscopy images showed the RUNX3 protein in green fluorescence and nuclei in blue fluorescence. The merged image of H_2_O_2_-treated cells revealed the cytosolic localization of RUNX3. DNA was stained with DAPI. **D,** **E **Western blot (WB) analysis was performed using antibodies against HDAC1, HAT1, EHMT2/G9a, MLL, and RUNX3. TBP was used as the loading control for nuclear protein fraction. **D** ^*^Significantly different from 0 h of H_2_O_2_ treatment group (*p* < 0.05). E ^*^Significantly different from control cells (*p* < 0.05); ^**^significantly different from H_2_O_2_-treated cells (*p* < 0.05). Cells were transfected with siHDAC1 RNA or siG9a RNA and treated with H_2_O_2_ for 48 h. **F** WB analysis for RUNX3 detection was performed. ^*^Significantly different from H_2_O_2_ treated-siControl cells (*p* < 0.05). **G **Confocal microscopy images showed the RUNX3 protein in green fluorescence, and nuclei in blue fluorescence. The merged image of siHDAC1 RNA- or siG9a RNA-transfected cells with H_2_O_2_ treatment indicated the nuclear localization of RUNX3

To find the optimal concentration of H_2_O_2_ treatment to study oxidative stress-induced RUNX3 mislocalization, we tested cell viability at various concentrations of the oxidative stressor H_2_O_2_. Cytotoxicity was not observed for concentrations of up to 100 μM H_2_O_2_ (Fig. 1A). Therefore, 100 μM H_2_O_2_ was considered the optimum concentration to induce RUNX3 mislocalization due to oxidative stress. 100 μM H_2_O_2_ increased the ROS levels, but a ROS scavenger, N-acetyl-l-cysteine (NAC), led to a reduction (Fig. 1B). Notably, H_2_O_2_ increased RUNX3 mislocalization from the nucleus to the cytoplasm; whereas NAC attenuated this effect (Fig. 1C).

It has been reported that oxidative stress induces the cytoplasmic localization of RUNX3 through HDAC1 [13, 18]. Therefore, to investigate whether histone modifications are involved in oxidative stress-induced RUNX3 mislocalization, the expression of histone modification-related proteins was evaluated. H_2_O_2_ treatment increased nuclear HDAC1 protein level in a time-dependent manner up to 12 h and decreased from 24 h, but still increased nuclear HDAC1 protein level to a level greater than that at 0 h (Fig. 1D). Furthermore, H_2_O_2_ treatment decreased nuclear histone acetyltransferase 1 (HAT1) protein level in a time-dependent manner (Fig. 1D) and increased G9a expression in the nucleus, but decreased the expression of MLL in a time-dependent manner (Fig. 1D). In addition, RUNX3 expression was opposite that of HDAC1 and G9a, in a time-dependent manner (Fig. 1D). Treatment with NAC inhibited the increased expression of HDAC1 and G9a induced by oxidative stress and reversed the reduced-RUNX3 expression by H_2_O_2_ treatment (Fig. 1E).

Therefore, to determine whether HDAC1 and G9a are important for the oxidative stress-induced mislocalization of RUNX3 from the nucleus to the cytoplasm, siRNAs targeting HDAC1 and G9a were used. Treatment with siHDAC1 RNA or siG9a RNA restored the nuclear localization of RUNX3, which was reduced by oxidative stress (Fig. 1F and G).

### HDAC1 and G9a Activate Src Following Oxidative Stress

**Fig. 2:**
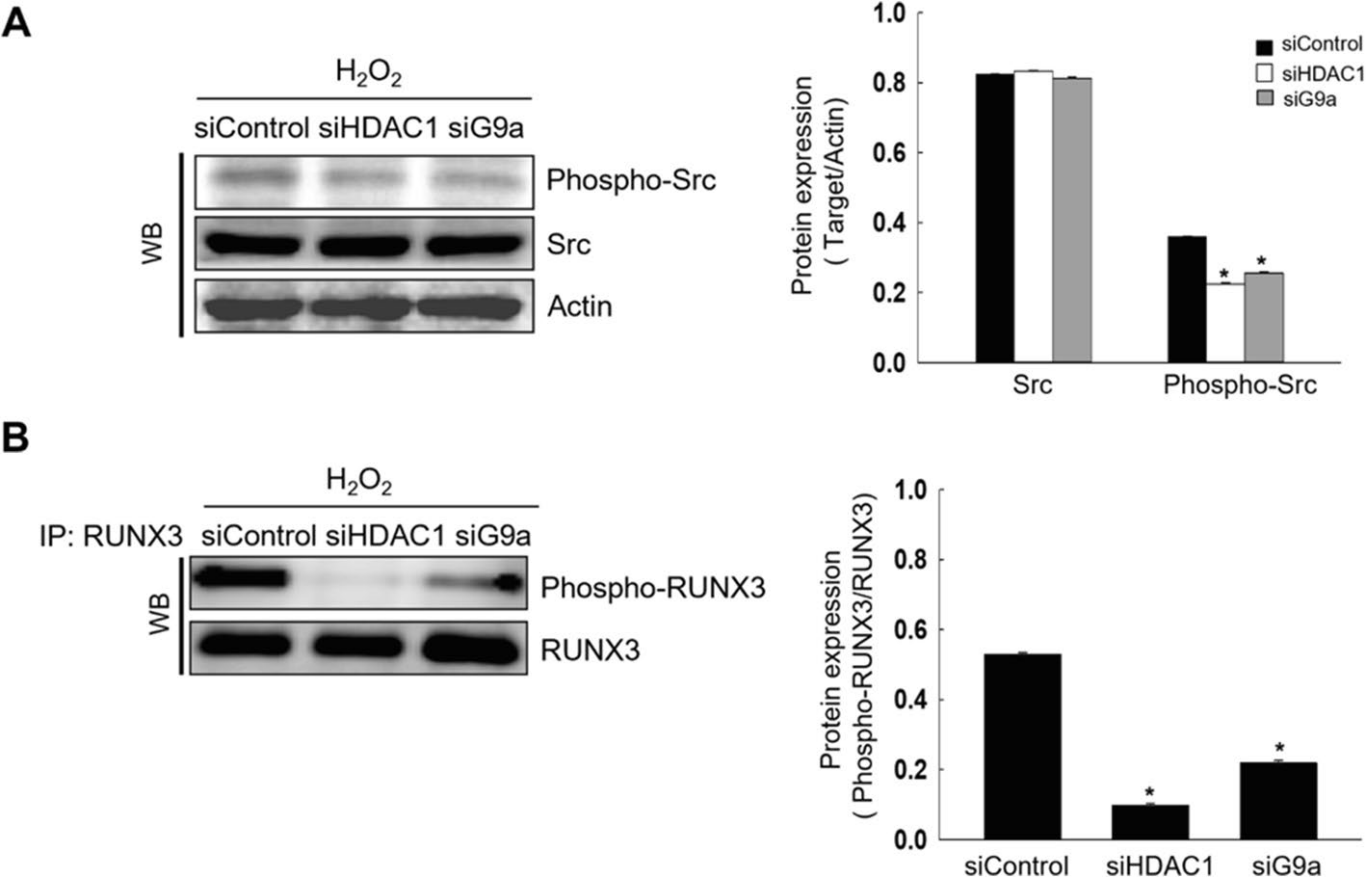
HDAC1 and G9a activate Src following oxidative stress. Cells were transfected with small interfering RNA HDAC1 or G9a and exposed to H_2_O_2_ for 48 h. **A** Levels of phospho-Src (the active form of Src) and Src were assessed using western blotting (WB). Actin was used as the loading control for total protein fraction. ^*^Significantly different from H_2_O_2_ treated-siControl cells (*p* < 0.05). **B** Cellular lysates were subjected to immunoprecipitation (IP) using antibody against RUNX3, and subsequently examined by WB using antibody against phospho-tyrosine (phospho-RUNX3). ^*^Significantly different from H_2_O_2_ treated-siControl cells (*p* < 0.05)

Our earlier study revealed that oxidative stress stimulates RUNX3 phosphorylation at tyrosine residues through Src activation, leading to mislocalization [13]. HDAC1 and G9a knockdown attenuated oxidative stress-induced Src and RUNX3 phosphorylation at tyrosine residues (Fig. 2A and B). These results suggested that oxidative stress-induced HDAC1 and G9a induced Src activation and led to RUNX3 phosphorylation, resulting in the cytoplasmic RUNX3 localization.

### Oxidative Stress Induces JAB1 and CRM1 Interaction Via HDAC1 Or G9a-Induced Src Activation

**Fig. 3:**
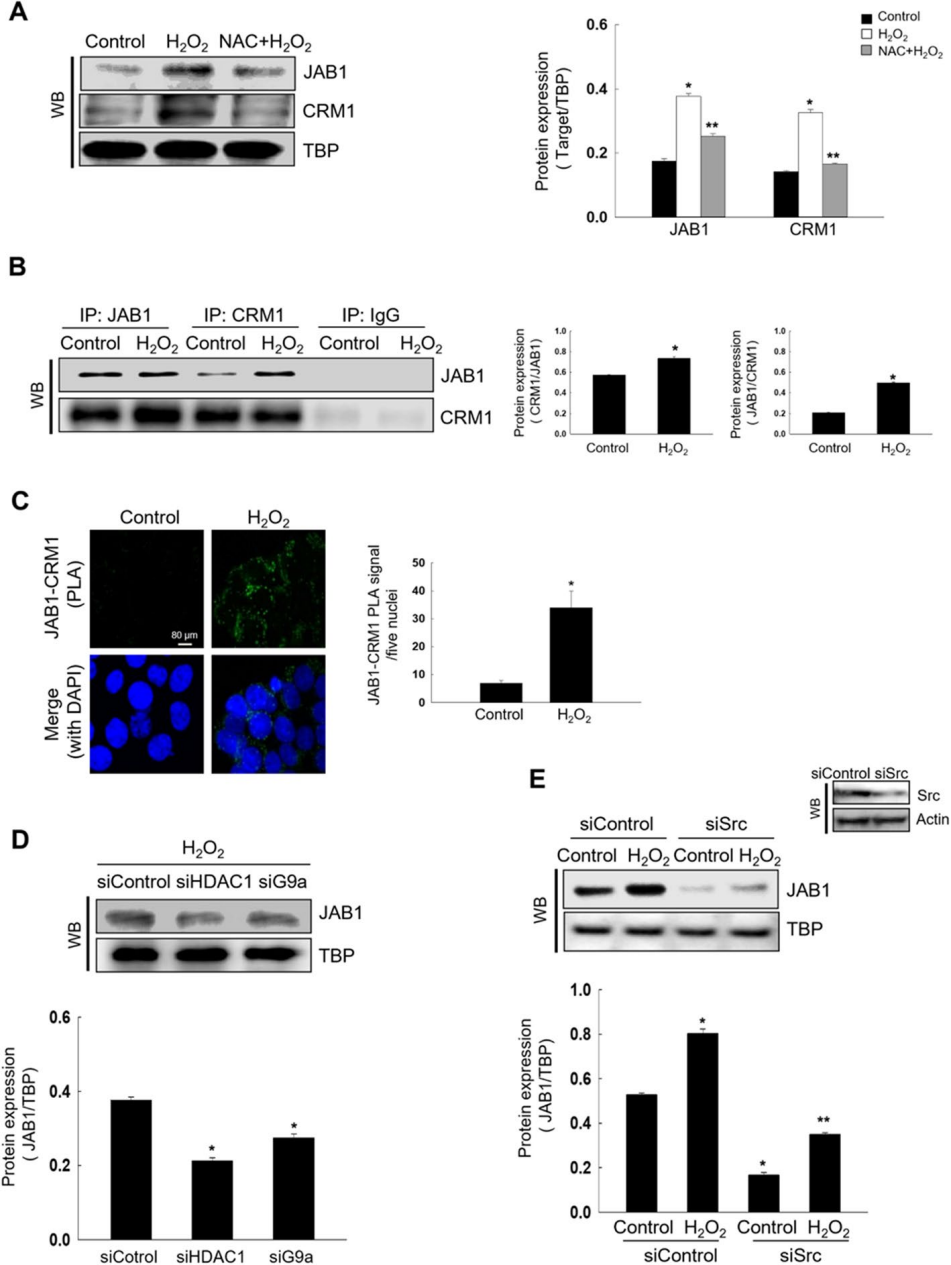
Oxidative stress induces JAB1 and CRM1 interaction via HDAC1 or G9a-induced Src activation. **A** Nuclear fraction was assessed using antibodies against JAB1, and CRM1. ^*^Significantly different from control cells (*p* < 0.05), ^**^significantly different from H_2_O_2_-treated cells (*p* < 0.05). **B** Cell lysates were immunoprecipitated with antibodies against JAB1 and CRM1 and subjected to WB. ^*^Significantly different from control cells (*p* < 0.05). **C** The interaction between JAB1 and CRM1 was assessed using PLA; each green spot represented a single interaction. DNA was stained with DAPI. ^*^Significantly different from control cells (*p* < 0.05). **D**, **E **WB was assessed for detection of nuclear JAB1 expression (**D**) in siHDAC1 RNA- or siG9a RNA-transfected cells with H_2_O_2_ treatment or (**E**) in Src-transfected cells with H_2_O_2_ treatment. **D** ^*^Significantly different from H_2_O_2_ treated-siControl cells (*p* < 0.05). **E** ^*^Significantly different from siControl cells (*p* < 0.05), ^**^Significantly different from H_2_O_2_ treated-siControl cells (*p* < 0.05)

JAB1 induces the cytoplasmic localization and degradation of RUNX3 [10, 11]. JAB1-mediated nuclear export requires CRM1, a member of the importin-related nuclear transport receptor family [24, 25]. Thus, we examined whether JAB1 is involved in oxidative stress-induced cytoplasmic RUNX3 localization. JAB1 and CRM1 expression increased in H_2_O_2_-treated cells; however, this effect was attenuated by NAC treatment (Fig. 3A). The interaction between JAB1 and CRM1 also increased in H_2_O_2_-treated cells, as determined by immunoprecipitation and PLA (Fig. 3B and C). Moreover, knockdown of HDAC1, G9a, and Src attenuated oxidative stress-induced JAB1 expression (Fig. 3D and E).

### Oxidative Stress Induces RUNX3 Mislocalization Via RUNX3, JAB1, and CRM1 Interaction

**Fig. 4:**
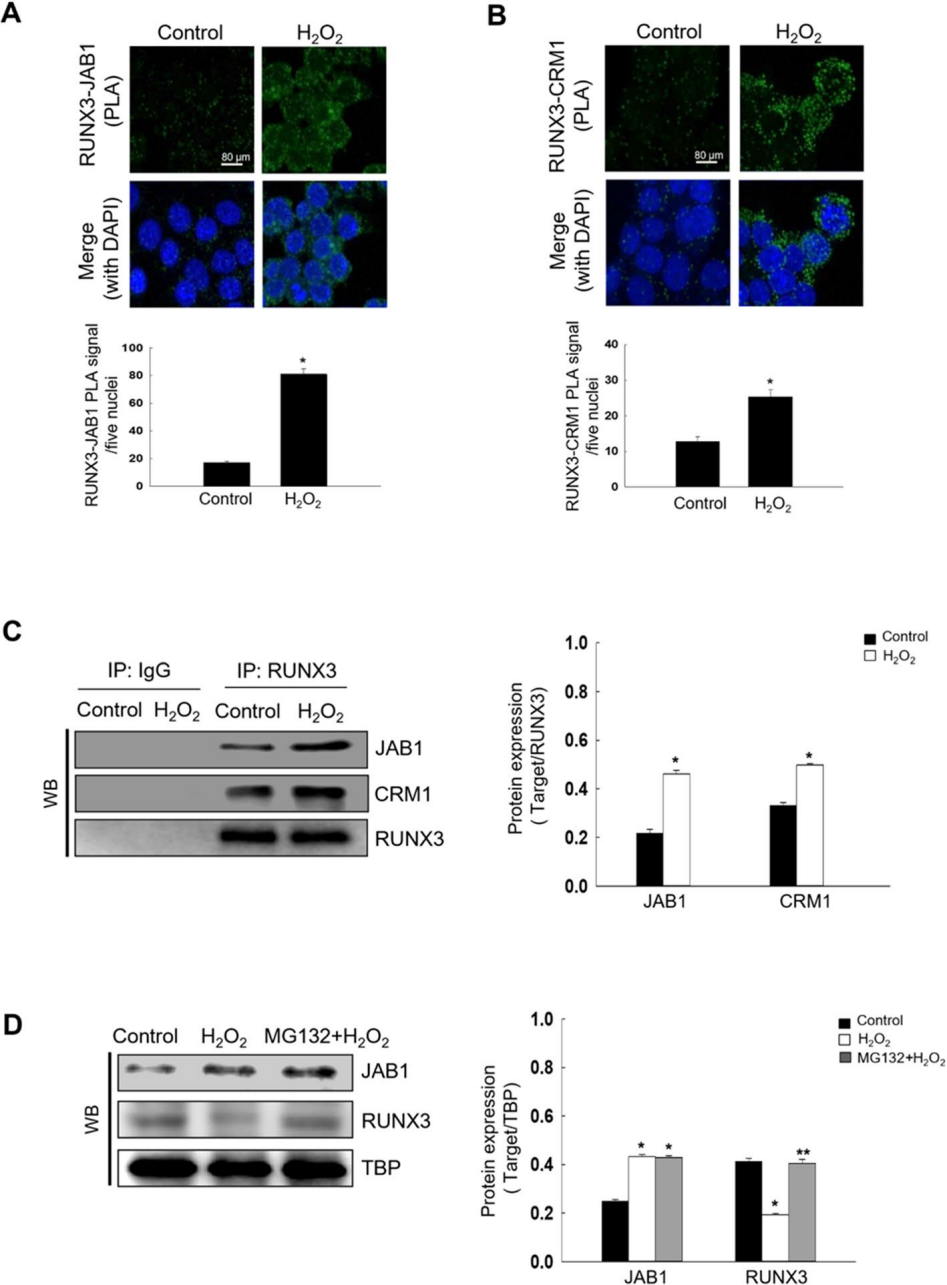

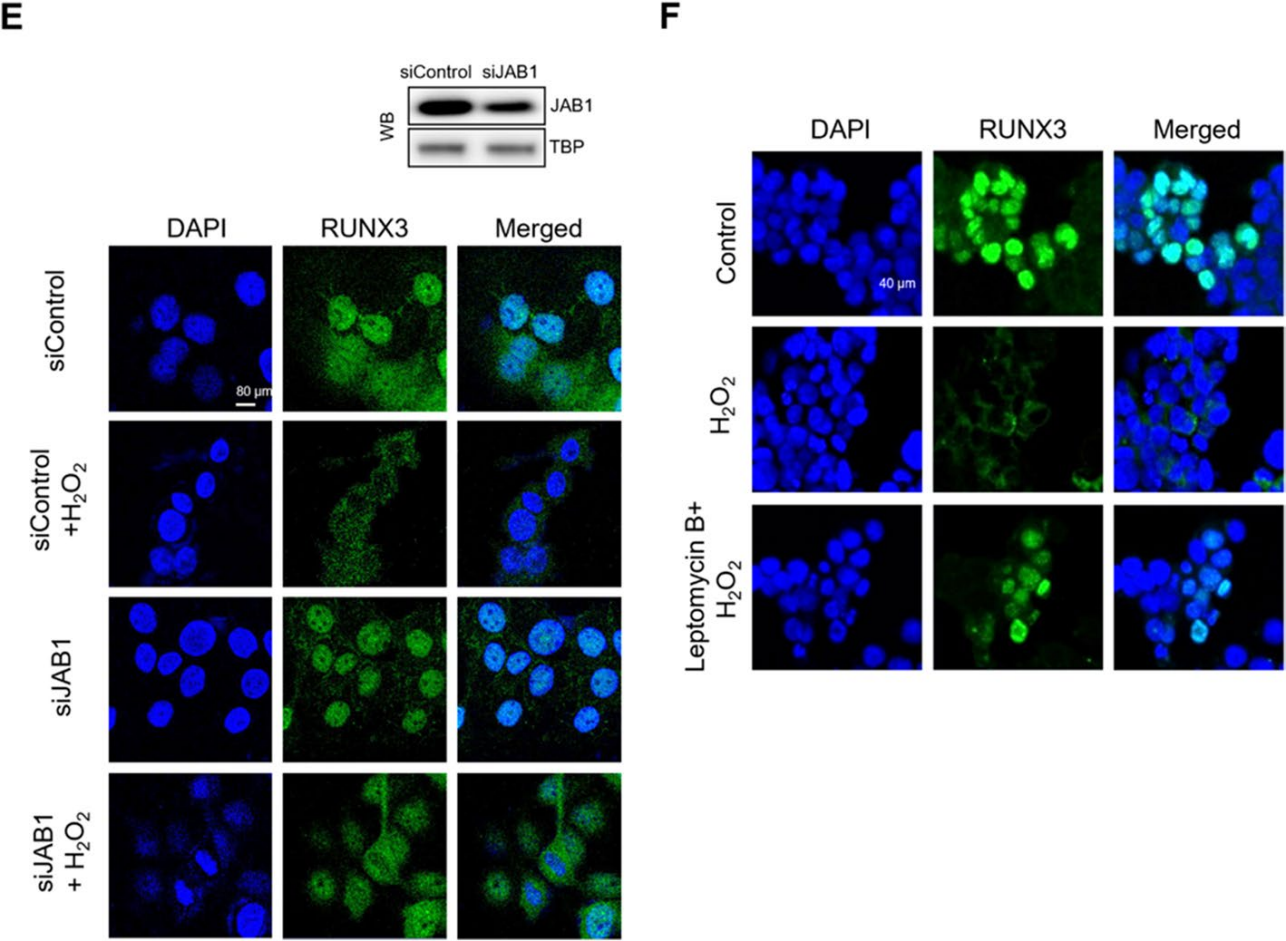
Oxidative stress induces RUNX3 mislocalization by inducing RUNX3, JAB1, and CRM1 interaction. **A**, **B** The interactions between (**A**) RUNX3 and JAB1 or (**B**) RUNX3 and CRM1 were assessed in H_2_O_2_-treated cells using PLA, and each green spot represented a single interaction. DNA was stained with DAPI. ^*^Significantly different from control cells (*p* < 0.05). **C** The interactions of RUNX3 and JAB1 or RUNX3 and CRM1 were examined using IP with an antibody against RUNX3, followed by WB with antibodies against JAB1, CRM1, and RUNX3. Cells were pretreated with 20 µM MG132 for 1 h and then treated with H_2_O_2_ for 48 h. ^*^Significantly different from control cells (*p* < 0.05). **D** Cell lysates were analyzed by WB with antibodies against JAB1 and RUNX3. Cells were transfected siJAB1 RNA or 10 nM leptomycin B and treated with H_2_O_2_ for 48 h. ^*^Significantly different from control cells (*p* < 0.05); ^**^significantly different from H_2_O_2_-treated cells (*p* < 0.05). **E**, **F** Confocal microscopy images showed RUNX3 protein in green fluorescence and nuclei in blue fluorescence. **E** The merged image of siJAB1 RNA-transfected cells with H_2_O_2_ treatment indicated the nuclear localization of RUNX3. **F** The merged image of leptomycin B-treated cells with H_2_O_2_ treatment indicates the nuclear localization of RUNX3

To determine whether the JAB1-mediated RUNX3 nuclear export is CRM1-dependent, we investigated the interactions of RUNX3 with JAB1 and CRM1. PLA and immunoprecipitation revealed that JAB1 and CRM1 interacted with RUNX3 in H_2_O_2_-treated cells (Fig. 4A-C). To investigate the JAB1-mediated degradation of RUNX3 induced by oxidative stress, we used the proteasome inhibitor, carbobenzoxyl-L-leucyl-L-leucyl-L-leucine (MG132). H_2_O_2_ treatment downregulated RUNX3 via JAB1 induction, however, RUNX3 downregulation was inhibited by MG132 (Fig. 4D). These data implied that JAB1 promoted RUNX3 degradation via the proteasome activity. In addition, JAB1 knockdown or treatment with a CRM1 inhibitor, leptomycin B, markedly increased the nuclear localization of RUNX3 in H_2_O_2_-treated cells (Fig. 4E and F). These data suggested that JAB1-mediated RUNX3 nuclear export required a CRM1-dependent pathway.

## Discussion

Oxidative imbalances that generate excessive ROS may play a direct or indirect role in the initiation, promotion, and development of carcinogenesis [26]. DNA and chromosomes are damaged by ROS, which can interact with oncogenes or tumor suppressor genes, induce epigenetic changes in DNA or histones, and alter immunological mechanisms [27, 28]. RUNX3 regulates cell proliferation, apoptosis, and differentiation. In various cancer cell types, RUNX3 is inactivated due to epigenetic silencing and cytoplasmic localization from the nucleus to the cytoplasm, thereby losing its function as a tumor suppressor [8]. Previously, we documented that oxidative stress promoteed RUNX3 mislocalization via HDAC1-induced Src activation and RUNX3 expression inhibition via DNA methylation in colon cancer cells [13, 19].

This study addressed the impact of oxidative stress on the unusual localization of RUNX3 in colon cancer cells and examined the role of histone modification-related proteins and nuclear exporters in this process. The results showed that under oxidative stress, the nuclear RUNX3 localization decreased and accumulated in the cytoplasm. We also found that the expression of the histone modifiers, HDAC1 and G9a, was induced by oxidative stress. In addition, the use of siRNAs to inhibit HDAC1 and G9a resulted in a reversal of the cytoplasmic localization of RUNX3, which was promoted by oxidative stress, suggesting that RUNX3 mislocalization was linked to histone deacetylation and methylation processes. As previously stated, oxidative stress promotes HDAC1 upregulation in colon cancer cells [13, 20]. HDAC1 and G9a overexpression inhibits the nuclear localization and RUNX3 expression under hypoxic condition in gastric cancer cells [18]. Under certain condition, hypoxia contributes to the formation of ROS [29]. Moreover, *H. pylori* infection promotes the mislocalization of p27 and decreases p27 expression by inhibiting histone acetylation within the p27 promoter [30].

Suberoylanilide hydroxamic acid, an HDAC inhibitor, effectively reduces the pro-oncogenic effect of the hepatitis B virus pre-S2 mutant oncoprotein in hepatocellular carcinoma cells and promotes the generation of the tumor suppressor protein thioredoxin-binding protein 2 (TBP2), which consequently strengthens the association between JAB1 and TBP2, and suppresses the degradation of p27 triggered by pre-S2 mutants [31, 32].

Src activation-mediated RUNX3 phosphorylation in *H. pylori-*infected intestines leads to the cytoplasmic localization of RUNX3 and causes intestinal metastasis [15]. In addition, *H. pylori*-induced ROS production in gastric epithelial cells can affect cellular signal transduction, leading to gastric carcinogenesis [33]. Our results showed that oxidative stress induces Src activation, which in turn phosphorylates the tyrosine residues of RUNX3 and induces cytosolic RUNX3 localization.

JAB1 is an essential component of the COP9 signalosome and is a multifunctional protein involved in integrin signaling, cell cycle regulation, and development [34, 35]. JAB1 is associated with the initiation and progression of numerous types of cancer, including hepatocellular carcinoma [35, 36]. The Mpr1/Pad1 N-terminal domain of JAB1 and the runt domain of RUNX3 physically interact; which enhances CRM1-dependent nuclear transportation of RUNX3 by JAB1. Furthermore, the sequestered cytosolic RUNX3 is subsequently eliminated by a process that depends upon the proteasome [10, 11]. Our data showed that JAB1 was activated by oxidative stress and that treatment with siHDAC1 RNA, siG9a RNA, and siSrc RNA inhibited JAB1 expression. JAB1 enhances the nuclear export of the tumor suppression proteins p27 and p53 by serving as a molecular mediator of CRM1, a carrier protein for nuclear export, and induces subsequent cytoplasmic degradation of these proteins [11, 37]. JAB1 and CRM1 interactions were induced and the cytoplasmic localization of RUNX3 was increased by oxidative stress. Treatment with siHDAC1 RNA, siG9a RNA, and siSrc RNA inhibited JAB1 expression, suggesting that the functional inhibition of RUNX3 during oxidative stress occurred, in part, via JAB1 and CRM1 mediated-mislocalization by HDAC1 or G9a-induced Src activation. Our findings demonstrated that the cytoplasmic distribution of RUNX3 in colon cancer cells under oxidative stress can be targeted for the development of novel therapy, as well as serving as potential biomarkers for the prediction of the occurrence of colon cancer.

## Data Availability

All data and materials in the manuscript will be made available to the corresponding authors.
